# Potent biological activity of newly fabricated silver nanoparticles coated by a carbon shell synthesized by electrical arc

**DOI:** 10.1038/s41598-024-54648-y

**Published:** 2024-03-04

**Authors:** Bassma H. Elwakil, Ahmed M. Eldrieny, Awatif Rashed Z. Almotairy, Mostafa El-Khatib

**Affiliations:** 1 Department of Medical Laboratory Technology, Faculty of Applied Health Sciences Technology, Pharos University in Alexandria, Alexandria, 21526 Egypt; 2 Department of Radiological Imaging, Faculty of Applied Health Sciences Technology, Pharos University in Alexandria, Alexandria, 21526 Egypt; 3 Department of Chemistry, Faculty of Science, Taibah University, 30799 Yanbu, Saudi Arabia; 4 Department of basic sciences, Faculty of Computer Science and Artificial Intelligence, Pharos University in Alexandria, Alexandria, 21526 Egypt

**Keywords:** Arc discharge, Carbon-silver nanoparticles, Molecular study, Confocal microscope, Biophysics, Microbiology, Diseases, Infectious diseases, Materials science, Nanoscale materials

## Abstract

Highly effective AgNPs@C was efficiently synthesized by electrical arc powered by single spark unit which was sufficient to ionize the dielectric media (deionized water) through applying strong electric field between the electrodes (silver and carbon). The AgNPs@C shell was characterized in terms of stability, morphology and phase structure. All characterizations showed that the prepared silver nanoparticles were spherical with average size reached 17 nm coated with carbon shell. The antibacterial effect of the synthesized nanoparticles was tested against *Pseudomonas aeruginosa* in comparison to Ceftazidime (commonly used antibiotic against *P. aeruginosa* infections). It was revealed that AgNPs@C shell has superior activity with inhibition zone diameter reached 15 mm and minimum inhibitory concentration reached 2 µg/mL. The observed activity was further confirmed by confocal microscope which showed an increased red region, representing the dead cells, correlated with the presence of AgNPs@C. Moreover, transmission electron microscope studies implied the possible AgNPs@C antibacterial mechanism of action was the nanoparticles adherence to the bacterial membrane causing cell lysis. The molecular studies against fimH (virulence adhesion gene), rmpA (mucoid factor encoding gene), and mrkA (biofilm forming gene) proved the inhibition of their genetic expression. The cytotoxic effect of the synthesized AgNPs@C showed CC50 reached 235.5 μg/mL against normal lung cells (L929 cell line).

## Introduction

Scientists have been competing in recent years to find ways to enhance the quality of life for human beings^1–6^. Nanomaterial applications play an important part in our daily lives, while their advantages and drawbacks can be either maximized or minimized. Nanomaterials' superior physical and chemical capabilities were due to their particles' size, shape, and composition^7^. Silver nanomaterials are among the most extensively synthesized materials^8^. There are three main categories that are utilized to produce silver nanoparticles (AgNPs) namely chemical, biological, and physical^9^. Physical synthesis has several advantages over the other two methods which can be summarized in the minimal impurity output specially upon using electrical arc discharge method^10^. Arc discharge is one of the oldest and most effective methods for producing high-quality carbon nanotubes along with other physical synthesis methods^1–10^. Changing the arc discharge settings offers a great deal of leeway for improving both quality and quantity. Arc current, power supply, temperature, pressure, medium, and electrodes shape are the most important factors in the arc discharge synthesis^11^. Direct current is often utilized for arc discharge while fewer studies were focused on the impact of alternating current (AC) versus pulsed power (PWM)^12,13^. On the other hand, extensive studies reported the impact of atmospheric part, and the correlations between pressure and the nanoparticle output^14–16^. It was reported that there is a direct correlation between the applied current and the size of the resulting nanoparticles^17,18^. Nanoparticles (NPs) in the arc discharge process are dependent on temperature for both nucleation and growth, and the relationship between these requires further research^19,20^. Moreover, electrodes’ geometry is a determining factor in the NPs production where smaller anode diameters leads to higher yields^21–23^. The use of organic solvents like acetone in the arc discharge technique leads to high average size nanoparticles, whereas deionized water yields a smaller and spherical product^24^. Further research is needed to determine the precise process underlying the nanoparticle creation^7,11^. AgNPs are not very stable in humid environments^25,26^. The key to successful silver nanoparticle preparation is preventing the development of aggregates. Several recent studies investigated the preparation of AgNPs as antimicrobial agent^27–30^. Strong stability will be indicated if the sample’s zeta potential was around ± 25.0 mV^10^, as shown in prior work, this can be achieved by adding a stabilizing agent such as polyvinylpyrrolidone (PVP) or sodium citrate^31,32^, which will extend the nanoparticles' shelf life from days to months.

In this work, AgNPs@C shells were produced, and their antibacterial activity were investigated against *Pseudomonas aeruginosa*. *Pseudomonas aeruginosa* was chosen due to its ranking among the highest global priority list of pathogens in 2017 according to World Health Organization (WHO)^33^. Moreover, it was ranked fifth among the most commonly isolated nosocomial pathogens in 28 European countries during 2016–2017^34^, while U.S. Centers for Disease Control and Prevention (CDC) reported that 32,600 hospitalized patients were infected by multi drug resistant (MDR) *P. aeruginosa* in 2017^35^. The increased resistance with newly emerged resistance mechanisms increased the threat of ESKAPE (*Enterococcus faecium*, *Staphylococcus aureus*, *Klebsiella pneumoniae*, *Acinetobacter baumannii*, *Pseudomonas aeruginosa*, and *Enterobacter* spp.) infections^36^. Multidrug-resistant (MDR)/extensively drug-resistant (XDR) *Pseudomonas aeruginosa* is a major threat with increased mortality, morbidity, and healthcare costs among the hospitalized patients^37^. In the context of antimicrobial management programmes, the treatment of these medical conditions is still difficult and calls for expert knowledge.

Hence, in the present study AgNPs@C was tested for its potential activity against *P. aeruginosa.* The fabrication technique relies heavily on high current and low voltage between the electrodes, which creates a strong electric field between the electrodes in the form of plasma. The use of an alternating current (AC) power source enables a fast and effective arc discharge procedure. The product was also characterized through transmission electron microscope (TEM), X-ray diffraction (XRD), Fourier-transform infrared spectroscopy (FTIR), Ultraviolet–visible (UV)-spectroscopy, and Energy dispersive X-ray (EDX).

## Materials and methods

### Materials

Silver rod (Purity 99%) was used as cathode submerged in deionized water (pH = 5.6, conductivity = 0.8–0.9 μS) with Carbon (Purity 99%) as anode. Whatman filter paper, Servo motor, arduino uno, potentiometers, multi speed motor, screw and pinion mechanism, monitor controller, electrodes holder, AC power supply, cooling system were used.

### Experimental system

**Figure 1:**
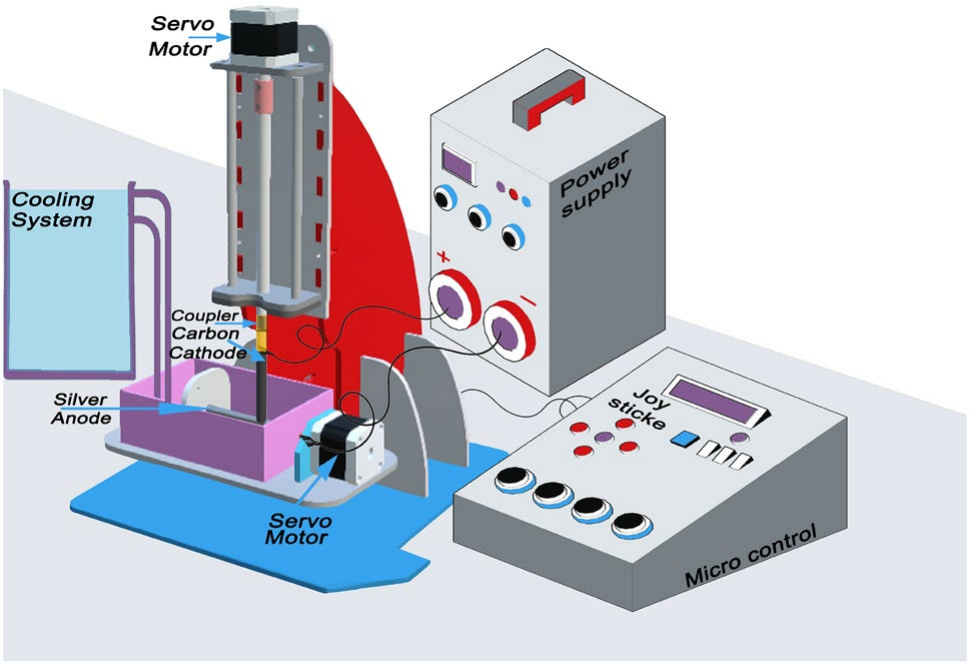
Electric arc discharge system^6^.

The system designed to create AgNPs@C shell shown in Fig. 1 composed of High purity silver cathode, microcontroller system, AC-power supply, an open vessel and motor. There are inlet and outlet holes for ethylene glycol’s constant flow rate supplying to maintain the temperature constant during the process.

Silver cathode has large dimensions in comparison to the carbon anode (Table 1) to increase the yield^10^. Each electrode was connected to a servo motor to maintain the gab constant. The rotational force accelerates clusters and prevents condensation on the electrode surfaces. The produced nanoparticles will be dispersed in the collecting medium (deionized water to yield fine shaped nanoparticles with small size)^21,22^ instead of trapping the produced particles on the electrode itself. The low voltage (30 V) and high current (15 A) produced by the AC power supply causes the silver electrode poles to vaporize or melt into silver clusters. The clusters are then quenched by the deionized water at 5 °C; eventually resulting in nanoparticles’ formation.

**Table 1:** The significantly important parameters used in the present arc discharge method.

Key parameters	Values
Discharging voltage (average value)	30 V
Discharging current (average values)	15 A
cathode disk (diameter, length)	16 mm, 10 cm
Anode (diameter, length)	2 mm, 5 cm
Discharging Duration time	10 min
Temperature of solution (before and After)	5^0^ C
Pressure	Atmospheric
PH	6
Volume of solution	3 L

The resulting yield were analyzed by UV–Vis spectrometer (Thermo Scientific™ Evolution 300), JEOL JEM-2100 high resolution transmission electron microscope, X-ray diffraction analyzer and Fourier Transform Infrared Spectroscopy (FTIR), and Energy dispersive X-ray (EDX) analysis. 

### Antibacterial activity

The antibacterial effect of the synthesized AgNPs@C was assessed through disc diffusion, minimum inhibitory concentration (MIC), minimum bactericidal concentration (MBC), bacterial lethality curve, quantitative real‐time PCR, live-dead fluorescent essay, and transmission electron microscope.

#### Disc diffusion assay

*Pseudomonas aeruginosa* optical density (OD) was adjusted to 0.1 OD. 25 µL of the adjusted bacterial suspension was inoculated on the Müeller-Hinton (MH) agar plates. While 25 µL of 50 μg/mL AgNPs@C suspension were loaded on sterile discs then the loaded discs were plated over the MH inoculated plates. Afterwards, the plates were incubated for 24 h at 37 °C^38^.

#### MIC and MBC determination

A serial dilution technique was used to determine the minimum concentration needed to inhibit bacterial growth according to Dev et al.^39^. After determining the MIC of the AgNPs@C, the dilution representing the MIC and five higher concentrations were plated on Müeller–Hinton agar plates then furtherly incubated for 24 h at 37 °C. The MBC endpoint was detected by checking pre and postincubated plates for the absence or presence of *P. aeruginosa* isolates growth^39^.

#### Bacterial lethality curve

The optimum time required to inhibit the bacterial growth was assessed through estimating the bacterial lethality curve. MIC value of the tested AgNPs@C was used throughout the present investigation. One mL of 1 × 10^6^ CFU/mL *P. aeruginosa* was added to 10 mL Müeller–Hinton broth containing AgNPs@C minimum inhibitory concentration then 100 µL were withdrawn to assess the bacterial growth at 0, 2, 4, 6, 8, 12 and 24 h (OD 600 nm)^40^.

#### RNA extraction and quantitative real-time PCR

Real-time PCR was used to examine the effect of AgNP@C on the fimH, rmpA, and mrkA genes’ expression (against the most resistant MDR *P. aeruginosa* strain). RNA was isolated by RNA extraction kit. Then the complementary DNA (cDNA) was synthesized using cDNA synthesis kit. 16S rRNA gene was utilized as a normalizing factor. To analyze the relative changes in gene expression ports, the 2 − ΔΔCt method was applied^41^.

#### Confocal scanning laser microscopy (CLSM)

The antibacterial effect of AgNPs@C was confirmed through confocal scanning laser microscopy [CLSM; Leica DMI 6000 B FluoView microscope (TCS SP5) coupled with confocal scanner (USA)]. Green fluorescence signal was 580-nm beam splitter employed with a long-pass 520-nm filter while red fluorescence signal was and long-pass 590-nm filter^42^.

#### Transmission electron microscope (TEM)

Further study through transmission electron microscope (JEM-100 CX Joel TEM) was applied to determine the possible antibacterial mechanism of action^38^.

### MTT essay

L929 normal lung cell line was used to determine the viability through 3‐(4,5‐ dimethylthiazol‐2‐yl)‐2,5‐diphenyltetrazolium bromide (MTT) assay. After 24 h of incubation (allowing the cell adherence), the cell medium was withdrew, and the cells were exposed to different concentrations (25, 50, 100, 200, and 400 µg/mL) of AgNPs@C. After 24 h of incubation, the medium in each well was replaced with MTT solution and fresh medium, and the cells were incubated for 4 h. Finally, the absorbance of each well was determined in a microtiter plate reader at 570 nm^43^.

### Institutional review board statement

This research work was approved for publication by unit of research ethics approval committee (UREAC), Faculty of Pharmacy, Pharos University in Alexandria (PUA/06/2023/4/30/3/084).

## Results and discussions

### Morphological examinations

**Figure 2:**
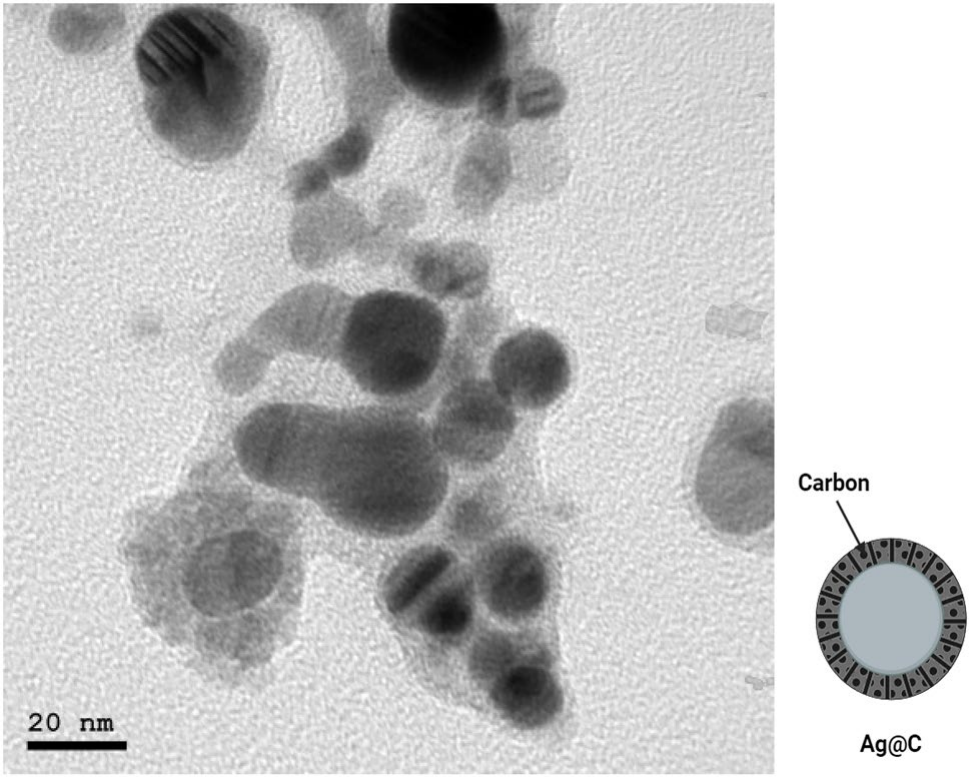
TEM of AgNPs@C shell.

TEM examinations were performed using JEOL JEM-2100 high resolution transmission electron microscope with an accelerating voltage of 200 kV. The obtained images showed silver nanoparticles with uniformed spherical shape which was in a good agreement with the face-centered cubic (FCC) Ag(111) planes, also the diameter of silver NPs was calculated by HR-TEM and found to be about 20 ± 3 nm. Figure 2 showed a dark sphere representing AgNPs coated with outer diameter about 3 nm of C shell. The preparation method influenced the yield where the light color C shell was functionalized and surrounding the dark spheres of the obtained AgNPs.

### Physicochemical characterization

#### XRD

**Figure 3:**
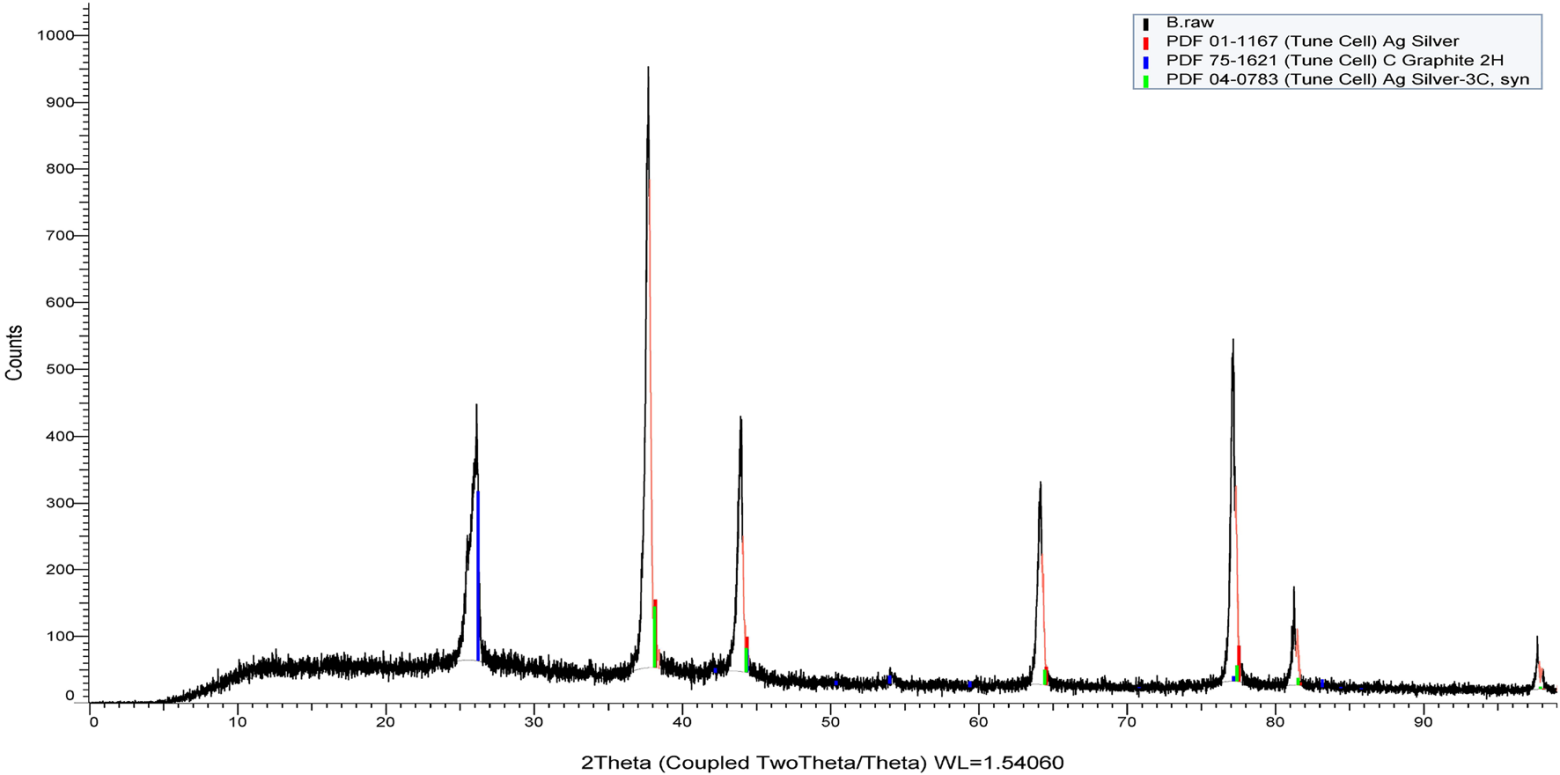
XRD pattren of Ag@C nanospheres.

The first peak at 2θ = 26.4° indicated the formation of carbon shell as assigned to the software with code JCPDS No. 01-0646^44^. The results were presented in Fig. 3. The obtained spectrum reveals the main peaks of silver phase at 2θ as tabulated in Table 2 which was in close agreement with the 04-0783^45^ standard card from the JCPDS. Average crystalline domain of AgNPs, determined by Debye–Scherrer formula, was approximately 17 nm for C and 26 ± 3 nm for AgNPs as detected by the XRD pattern. AgNPs and C diameters in the diffraction pattern were correlated with the average crystal size in the sample. This correlation was described by the Scherrer equation as shown in Eq. (1):

1 $${\text{D}} = {\text{K}}\uplambda /\left( {{\text{B}}\,{\text{cos}}\,\uptheta } \right)$$

where D is the crystal diameter, B is the full width at half maximum of the peak, θ represents the Bragg angle, and k is a constant related to the crystalline shape.

**Table 2:** XRD diffraction pattern of Ag@C nanospheres.

Diffraction angles (2θ) degree	d (Å) XRD	(h k l) Miller indices	Grain size (nm)	Particle type
26.228	3.39500	(002)	17 nm	CNTs
38.115	92	(111)	23 nm	AgNPs
44.299	37	(200)	25 nm	
64.443	23	(220)	28 nm	
77.397	24	(311)	28 nm	

The crystallite index of AgNPs was also calculated using the Scherrer method which was found to be in the range of 76% (polycrystalline) for Nano sized synthesized Ag@C. The crystallinity index was evaluated through the equation expressed as blew in Eq. (2):

2 $${\text{I}}_{{{\text{cry}}}} = {\text{D}}_{{\text{p}}} \left( {{\text{TEM}}} \right)/{\text{D}}_{{{\text{cry}}}} \left( {{\text{XRD}}} \right)$$

#### FTIR-analysis

**Figure 4:**
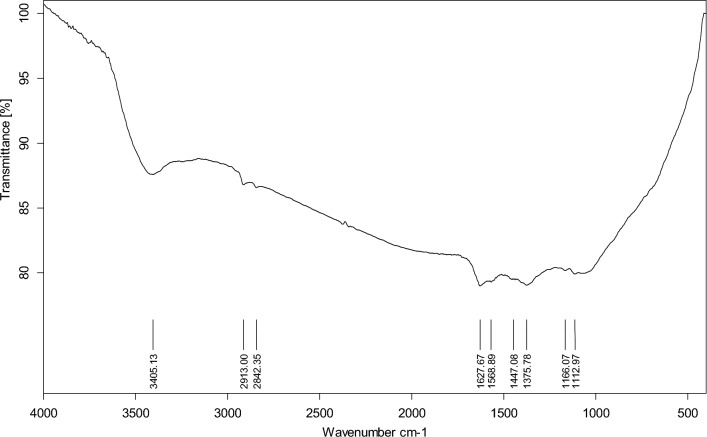
FTIR spectra of AgNPs@C nanospheres.

Figure 4 displayed the FTIR spectra of the AgNPs@C core shell. The strong and widespread absorption peak seen at 3405 cm^−1^ was created by the stretching vibration of OH^8^. The bands in the 2913–2842 cm^−1^ region could be attributed to the C–H stretching vibration^46^. The peaks at 1627 cm^−1^ and 1568 cm^−1^ were representative peaks of C=O (C_6_H_5_–C=O) and C=C vibrations, respectively^8^. Besides, the peaks from 1112 to 1447 cm^−1^ corresponded to the C–OH stretching and OH bending vibrations, which suggested the existence of many residual hydroxyl groups on the surfaces of AgNPs@C nanospheres^8,47^.

#### UV–vis analysis

**Figure 5:**
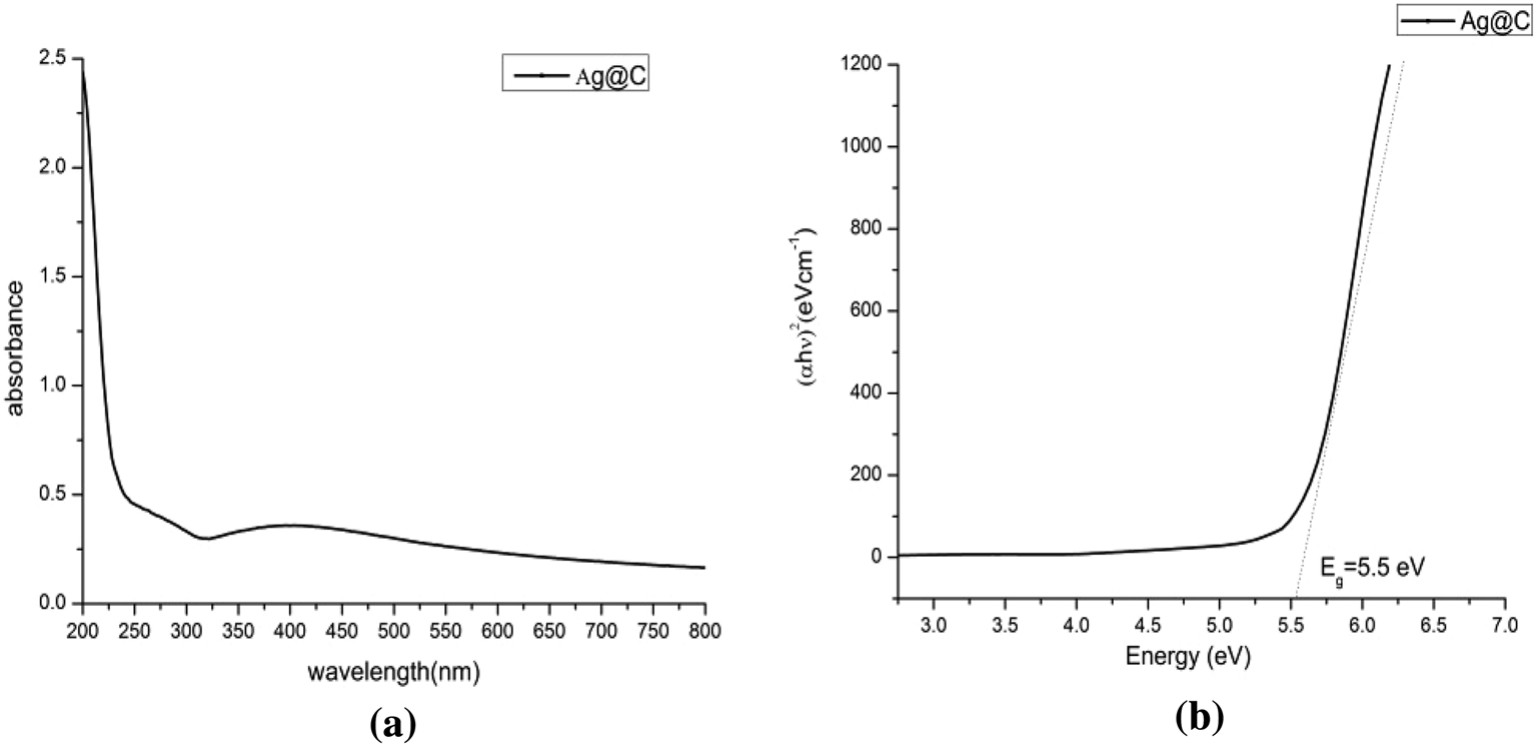
UV analysis (**a**) and band gap energy (**b**) of the fabricated AgNPs@C.

The sensitivity of UV–Vis is highly dependent on the shape of the nanoparticles that was indicated with a broad smooth peak around 396 nm that proved the formation of spherical shape silver nanoparticles (Fig. 5a) (probably a fairly wide absorbance band of silver due to excitation of electrons from the valence band to the conduction band). Another peak started around 200 nm refer to the presence of amorphous carbon. Moreover, owing to quantum confinement^48^, the optical energy band gap (Eg, eV) value of Ag@C NPs was 5.5 eV (Fig. 5b). However, no absorption peak of impurities was detected by using UV–vis spectroscopy. These results evidently indicate that Arc discharge technique are conspicuously successfully produced AgNPs@C without chemical impurities which can be a strength point in the medical applications^49^.

#### Energy dispersive X-ray (EDX)

**Figure 6:**
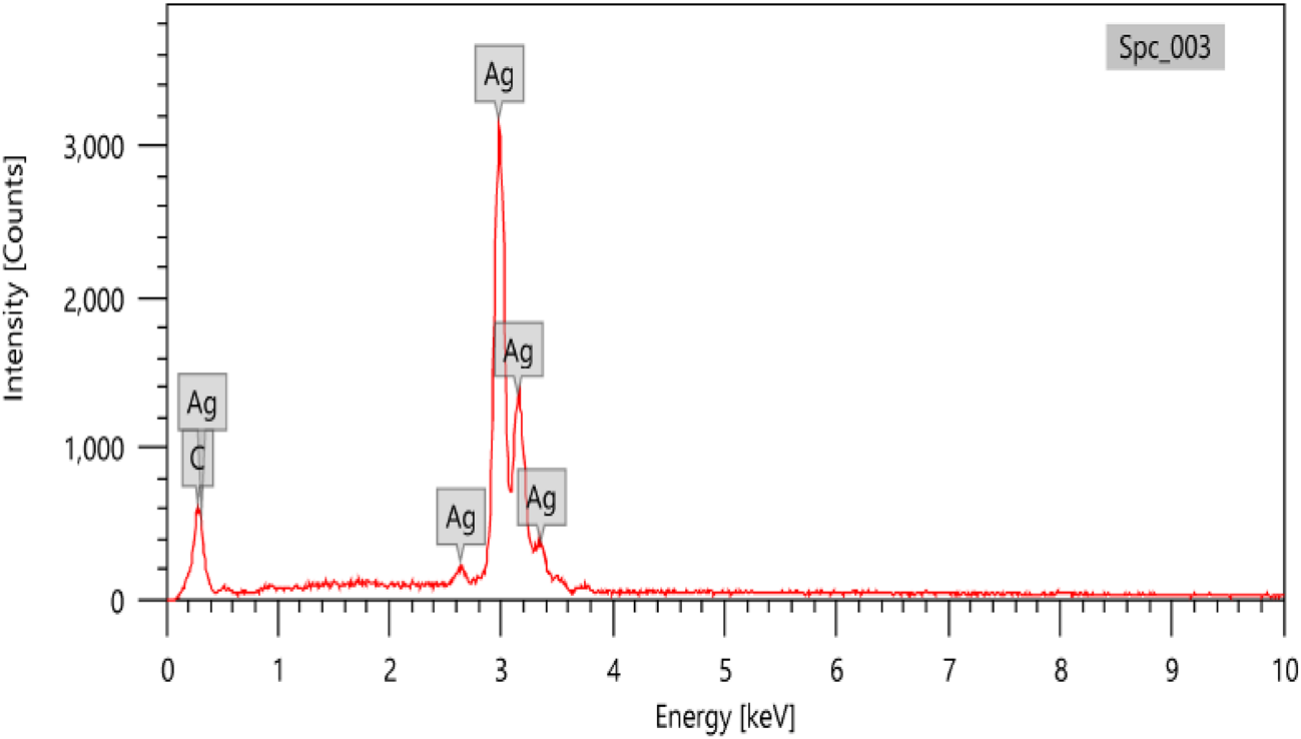
EDX analysis of the formulated Ag@C nanospheres.

Chemical purity of the product was examined by EDX for identification of elemental compositions of the yield which proved high purity of the sample ~ 100% as shown in Fig. 6. It was observed that the majority of the product was due to the presence of silver nanoparticles (59.29 ± 0.38 Atom%) while carbon Atom% was found to be 40.71 ± 0.50.

#### AgNPs@C stability

The type of capping agent, the surrounding environmental variables, such as pH, ionic strength, and the background electrolyte composition, all play a role in colloidal stability. If the NPs are not shielded by a capping agent that provides colloidal stability by electrostatic or steric repulsion, their high surface area to volume ratio results in high reactivity, which causes particle aggregation and settling. According to the traditional Derjaguin, Landau, Verwey, and Overbeek (DLVO) hypothesis, colloidal particles are encircled by a diffuse electrostatic double layer (EDL), and the equilibrium between the electrostatic repulsion and van der Waals forces controls the stability of the colloidal suspension. Solution characteristics like pH, ionic strength, and electrolyte ion valence are strongly related to the size of the electrical charge inside and the thickness of the EDL^50^. Hence The stability of the synthesized AgNPs@C was evaluated by monitoring the zeta potential changes in serum, phosphate buffer solution at pH 4, 7 and 10. Table 3 proved that AgNPs@C were highly stable at physiological pH and in serum. During the different incubation periods, the zeta potential measurements slightly increased with time. Generally, a suspension that exhibits a zeta potential less than ± 20 mV is usually considered unstable and will result in particles settling out of solution in the absence of other factors^50^.

**Table 3:** Zeta potential (mV) of AgNPs@C.

	24 h	48 h	72 h
PBS pH 4	− 30.4	− 36.7	− 40.1
PBS pH 7	− 25.0	− 25.4	− 26.0
PBS pH 10	− 43.8	− 46.9	− 48.5
Serum	− 27.1	− 27.8	− 28.0

### Antibacterial activity

**Figure 7:**
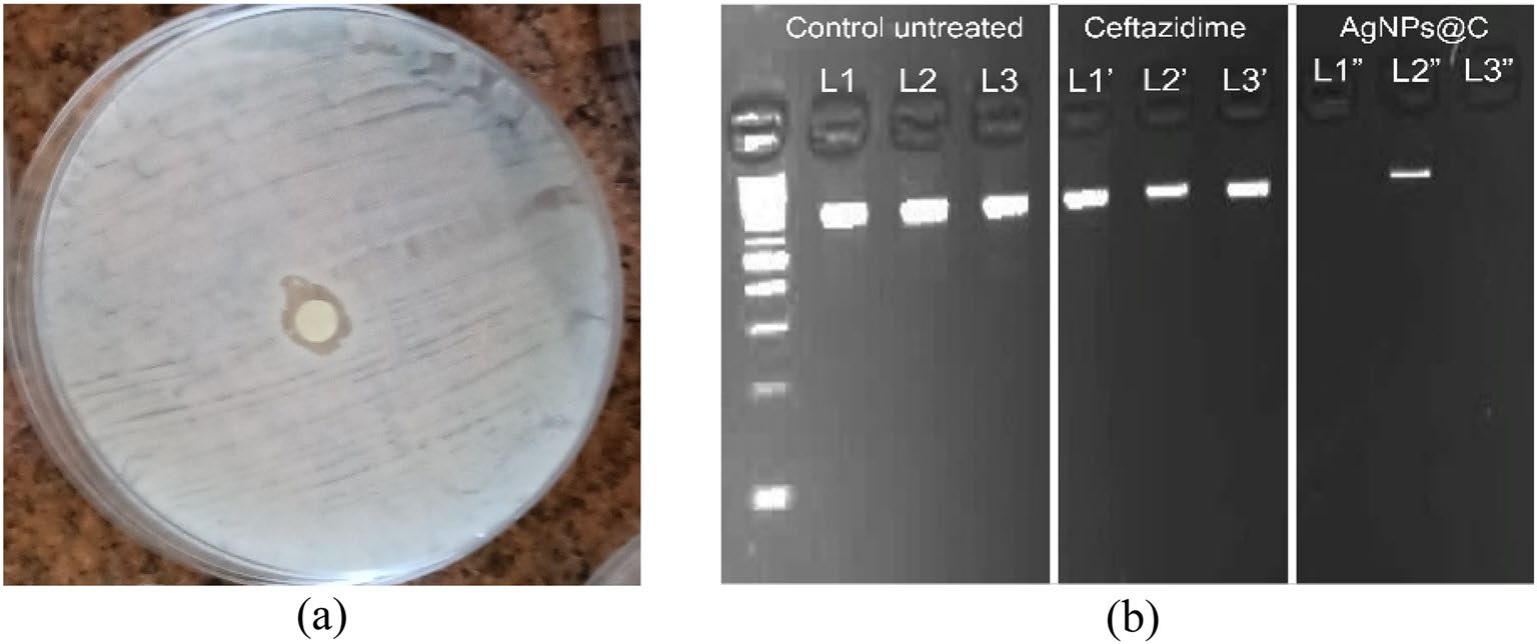
AgNPs@C activity assessment through disc diffusion method (**a**) and gene expression of fimH (L1, L1’and L1″), rmpA (L2, L2’and L2″), and mrkA (L3, L3’and L3″) genes expression (**b**).

**Figure 8:**
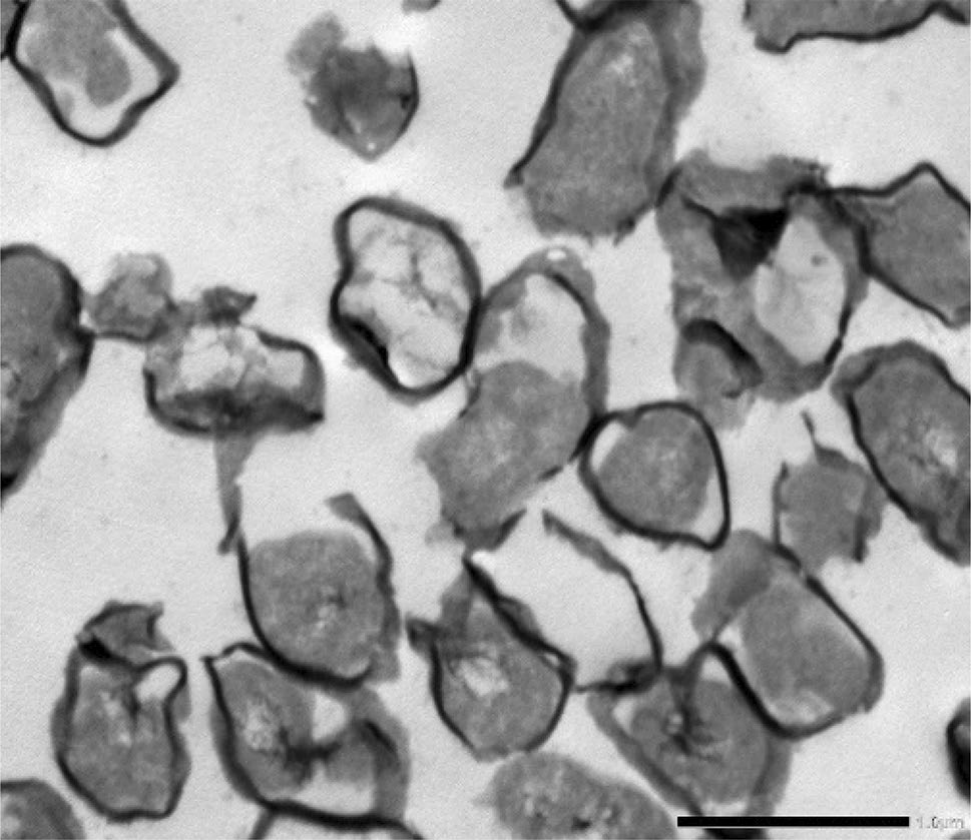
Transmission electron microscope study of P. aeruginosa treated cells.

**Figure 9:**
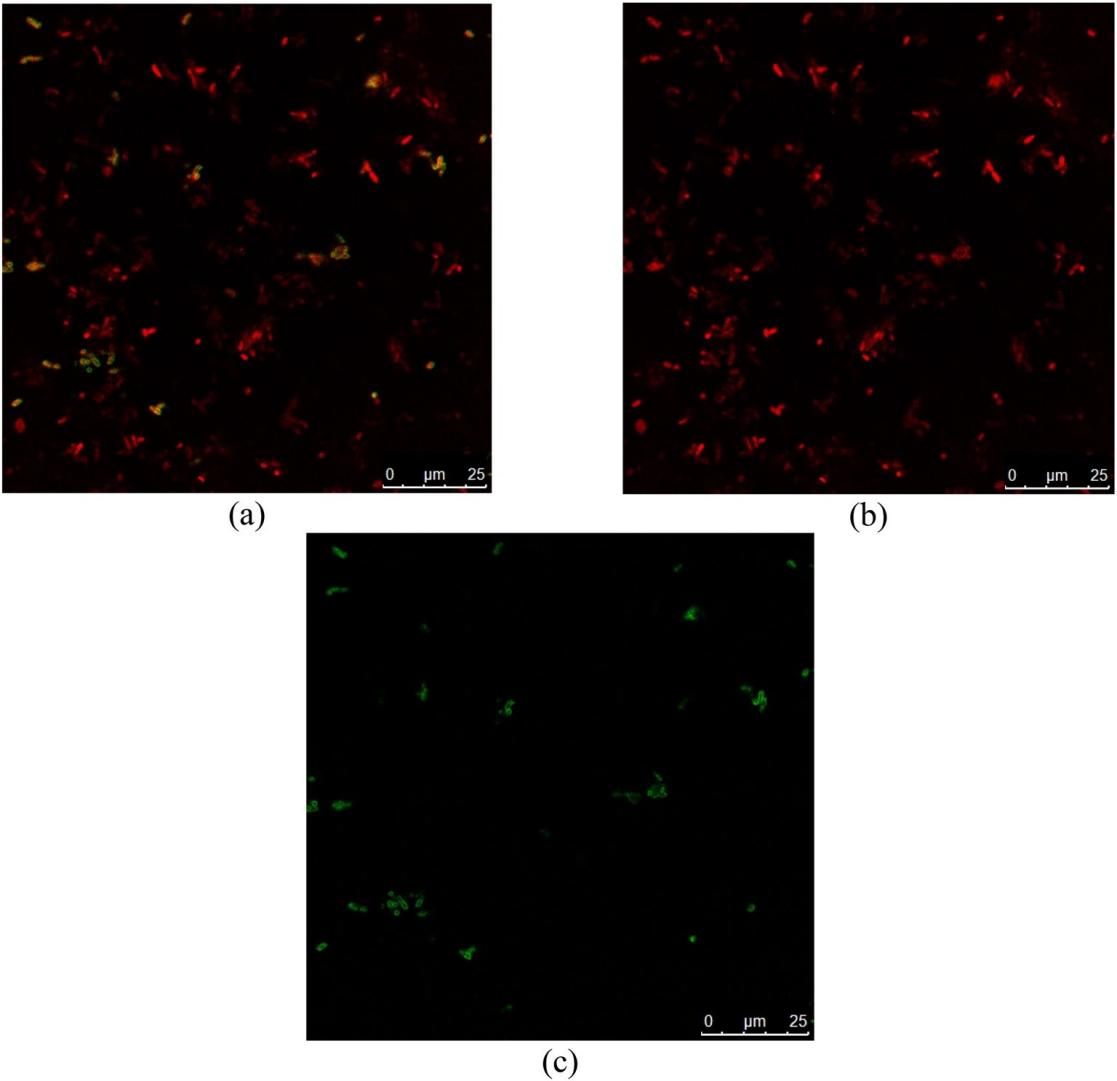
Confocal microscope study of bacterial treated cells where (**a**) merged, (**b**) dead and (**c**) viable cells.

Antibacterial Activity of AgNPs@C against *P. aeruginosa* was assessed through disc diffusion method, MIC, MBC, confocal laser microscope, molecular study, and transmission electron microscope examination. Disc diffusion method revealed that the synthesized AgNPs@C could inhibit the bacterial (*P. aeruginosa*) growth at a concentration equaled 50 µg/mL (Fig. 7a). Hence, MIC and MBC studies were required to assess the exact required concentration that would inhibit the bacterial growth. It was revealed that *P. aeruginosa* 2, *P. aeruginosa* 3 and *P. aeruginosa* 4 were the most resistant strains with MIC values reached 4, 4 and 16 µg/mL respectively (Table 4). These results were confirmed using the live and dead staining method, where red indicates the dead cells and green represents the live cells^51^. The live and dead assay of nanoparticles against *P. aeruginosa* were displayed in Fig. 9, which were in alignment with the plate assay results. These pictures showed an increasing red region, representing the dead cells, correlated with the presence of AgNPs@C. Thus, confirmed the cells death at the MIC dose of AgNPs@C. Transmission electron microscope study proved the bactericidal effect of the synthesized AgNPs@C (Fig. 8) where AgNPs@C attached to the bacterial membrane due to their surface negative charge causing cell lysis. Similar antibacterial mechanisms were reported by Xu et al.^52^ and Ssekatawa et al.^53^. Moreover, Barbalinardo et al.^54^ reported that the small spherical shape of silver nanoparticles was another possibility of the easy uptake and bacterial cell damage. It is worth noting that previous study stated that all of the surface parameters were responsible for the activity of the fabricated AgNPs against pathogens due to cytoplasmic cell wall membrane penetration leading to destroying the selective permeability, and eventually causing cell death^55^.

**Table 4:** Antibacterial activity of AgNPs@C.

Tested pathogen	Ceftazidime (antibiotic)			AgNPs@C		
	IZD (mm)	MIC (µg/mL)	MBC (µg/mL)	IZD (mm)	MIC (µg/mL)	MBC (µg/mL)
*P. aeruginosa* 1	10.0	8.0	64.0	10.0	2.0	16.0
*P. aeruginosa* 2	6.0	16.0	250.0	9.0	4.0	64.0
*P. aeruginosa* 3	6.0	16.0	250.0	9.0	4.0	64.0
*P. aeruginosa* 4	6.0	32.0	500.0	8.0	16.0	128.0
*P. aeruginosa* 5	11.0	8.0	128.0	15.0	2.0	32.0
*P. aeruginosa* 6	12.0	8.0	128.0	15.0	2.0	32.0

*IZD* inhibition zone diameter, *MIC* minimum inhibitory concentration, *MBC* minimum bactericidal concentration.

Another silver nanoparticles antibacterial mechanism of action was attributed to Ag^+^ ions release that intrude cell cycle, make the mitochondria malfunction and induce apoptosis in bacterial cells^56,57^. Several studies revealed that silver nanoparticles not only cause cell lysis but also increased ROS production, DNA damage, inhibition of proteins and ribosomes functions, etc.^58,59^. Hence in the present investigation it was important to assess the genetic damage caused by the synthesized nanoparticles. Data represented in Fig. 7b revealed the potent effect of the AgNPs@C against fimH (virulence adhesion gene), rmpA (mucoid factor encoding gene), and mrkA (biofilm forming gene) genetic expression which prove that the observed inhibitory effect occurred by intracellular signaling pathway hindrance including inhibition of both the bacterial ability to adhere to the host cell and biofilm formation (Figs. 8, 9).

### AgNPs@C cytotoxicity

**Figure 10:**
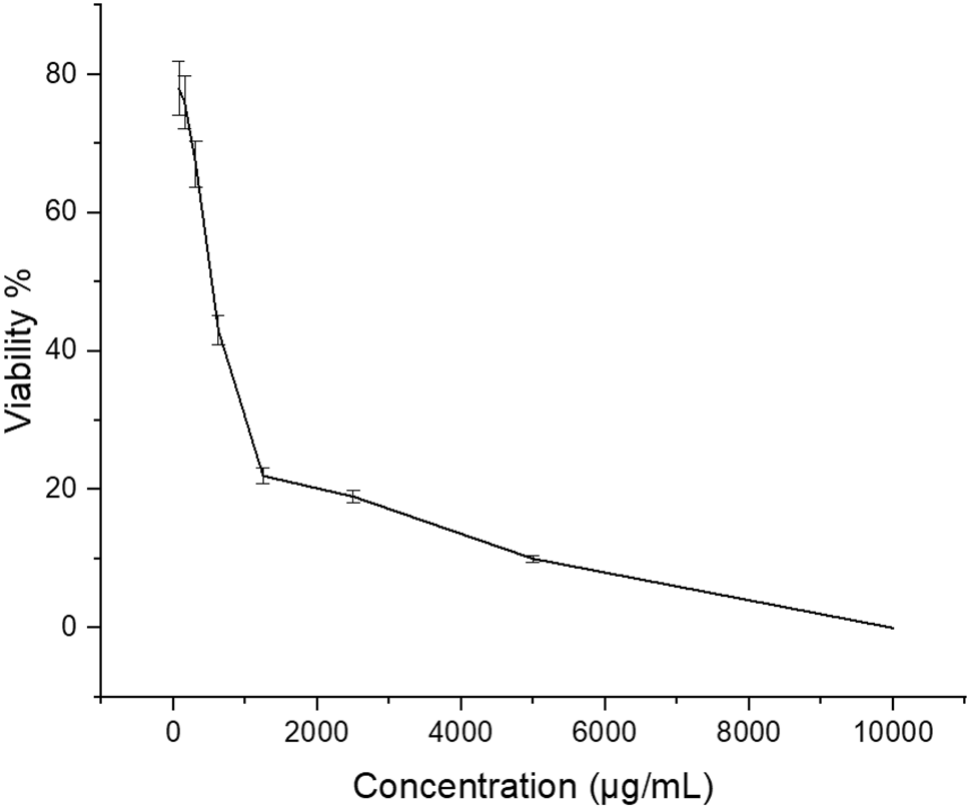
MTT essay.

The in vitro cytotoxic effect of the synthesized AgNPs@C was evaluated in L929 cell line. It was revealed that at 310 μg/mL of the tested nanoparticles, the normal lung cell viability% was 40% (Fig. 10). Moreover, data revealed that AgNPs@C showed CC50 reached 235.5 μg/mL. Hafez et al.^60^ found that despite the high antimicrobial activity of the prepared AgNPs@poly(tannic acid) (PTA) it showed high cytotoxicity towards L929 cells which hindered their biological applications and they needed to add natural cell viability enhancers (e.g. tannic acid and chitosan) to the prepared AgNPs@PTA (1000 ppm) in order to improve the cell viability.

## Conclusion

AgNPs@C synthesized by arc discharge method was highly stable, spherical with size 17 nm coated by carbon shell. The percentage of carbon mass increased when replacement carbon rod as anode compared with previous work^6^. Moreover results appear of more sharpness XRD of carbon peak of high intense peak at 2θ 26° indicate more coating of carbon to silver nanoparticles. The antibacterial effect of the synthesized nanoparticles was tested against *Pseudomonas aeruginosa* in relation to a commonly used antibiotic Ceftazidime. It was revealed that AgNPs@C shell showed superior activity over the used antibiotic. Confocal scanning fluorescent microscope and transmission electron microscope proved the bactericidal effect of the synthesized nanoparticles. On the other hand, the molecular studies of the genetic expression of fimH (virulence adhesion gene), rmpA (mucoid factor encoding gene), and mrkA (biofilm forming gene) in the presence of AgNPs@C implied that the observed antibacterial effect may be attributed to the intracellular signaling pathway hindrance including inhibition of both the bacterial ability to adhere to the host cell and biofilm formation. The cytotoxic effect of the synthesized AgNPs@C showed CC50 reached 235.5 μg/mL against normal lung cells (L929 cell line).

### Supplementary Information

Supplementary Information 1.

## Data Availability

All the original data are available upon reasonable request for correspondence authors.
