# Supplementary figures and images for: Potent biological activity of newly fabricated silver nanoparticles coated by a carbon shell synthesized by electrical arc
Source: Sci Rep. 2024 Mar 4;14:5324. doi: 10.1038/s41598-024-54648-y (PMC10912099; doi:10.1038/s41598-024-54648-y)

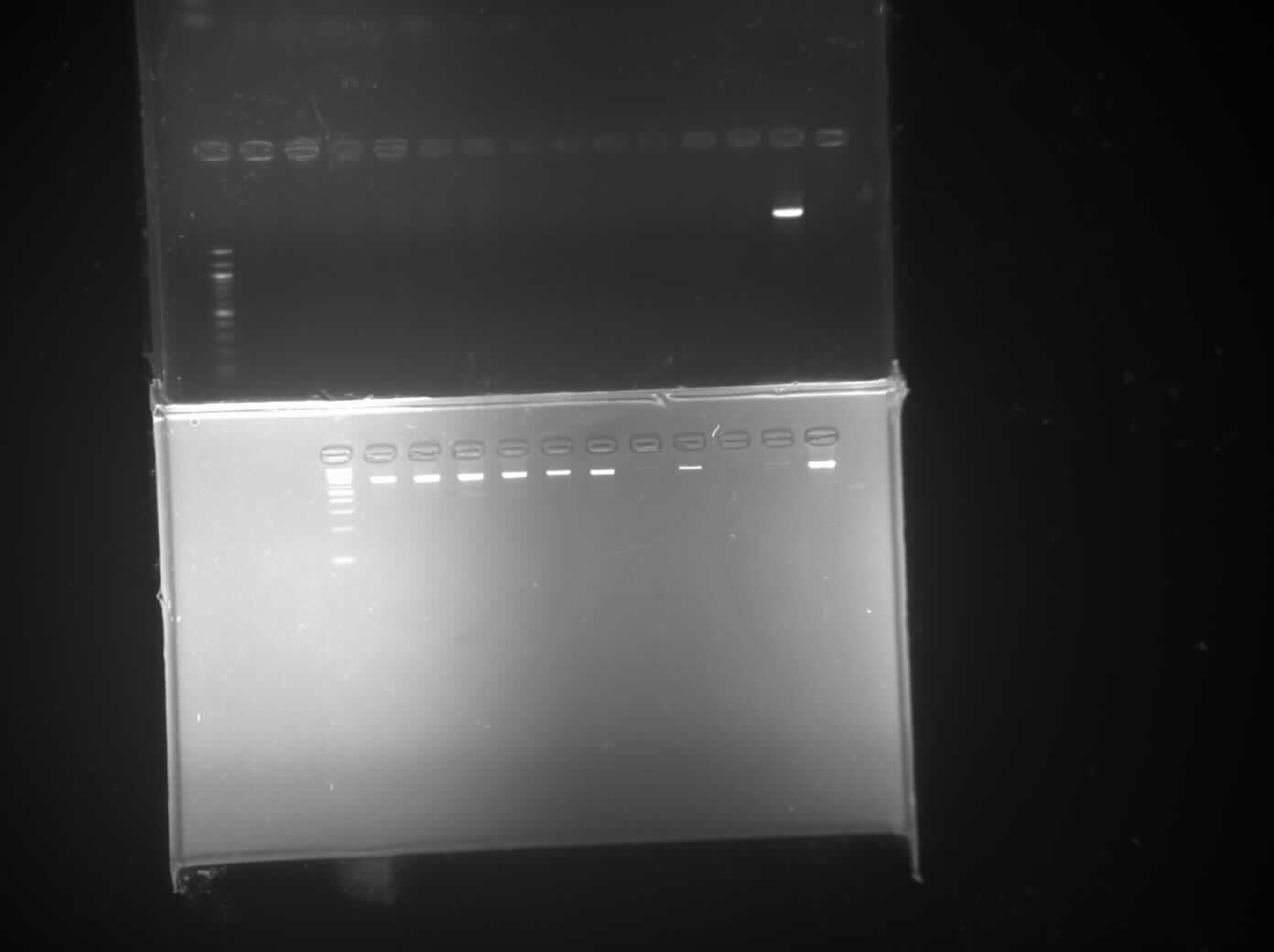

Supplement: Supplementary file 1 — Supplementary Information 1. [file 41598_2024_54648_MOESM1_ESM.jpg]
